# Engineering of xylose reductase and overexpression of xylitol dehydrogenase and xylulokinase improves xylose alcoholic fermentation in the thermotolerant yeast *Hansenula polymorpha*

**DOI:** 10.1186/1475-2859-7-21

**Published:** 2008-07-23

**Authors:** Olena V Dmytruk, Kostyantyn V Dmytruk, Charles A Abbas, Andriy Y Voronovsky, Andriy A Sibirny

**Affiliations:** 1 Institute of Cell Biology, NAS of Ukraine, Drahomanov Street 14/16, Lviv 79005, Ukraine; 2 Archer Daniels Midland Co Research Center, 1001 N Brush College Rd., Decatur IL 62521, USA; 3 Department of Microbiology and Biotechnology, Rzeszow University, Cwiklinskiej 2, 35-601, Rzeszow, Poland

## Abstract

**Background:**

The thermotolerant methylotrophic yeast *Hansenula polymorpha *is capable of alcoholic fermentation of xylose at elevated temperatures (45 – 48°C). Such property of this yeast defines it as a good candidate for the development of an efficient process for simultaneous saccharification and fermentation. However, to be economically viable, the main characteristics of xylose fermentation of *H. polymorpha *have to be improved.

**Results:**

Site-specific mutagenesis of *H. polymorpha XYL1 *gene encoding xylose reductase was carried out to decrease affinity of this enzyme toward NADPH. The modified version of *XYL1 *gene under control of the strong constitutive *HpGAP *promoter was overexpressed on a *Δxyl1 *background. This resulted in significant increase in the K_M _for NADPH in the mutated xylose reductase (K341 → R N343 → D), while K_M _for NADH remained nearly unchanged. The recombinant *H. polymorpha *strain overexpressing the mutated enzyme together with native xylitol dehydrogenase and xylulokinase on *Δxyl1 *background was constructed. Xylose consumption, ethanol and xylitol production by the constructed strain were determined for high-temperature xylose fermentation at 48°C. A significant increase in ethanol productivity (up to 7.3 times) was shown in this recombinant strain as compared with the wild type strain. Moreover, the xylitol production by the recombinant strain was reduced considerably to 0.9 mg × (L × h)^-1 ^as compared to 4.2 mg × (L × h)^-1 ^for the wild type strain.

**Conclusion:**

Recombinant strains of *H. polymorpha *engineered for improved xylose utilization are described in the present work. These strains show a significant increase in ethanol productivity with simultaneous reduction in the production of xylitol during high-temperature xylose fermentation.

## Background

Fuel ethanol produced from lignocellulosics is an environmentally friendly alternative liquid fuel to petroleum derived transportation liquid fuels. Xylose represents a significant constituent of the hemicellulose xylan fraction of lignocellulosics. Due to this, it is necessary to ferment xylose efficiently to ethanol to improve process economics for the production of this renewable liquid fuel [1].

Some yeasts, filamentous fungi and bacteria are able to convert xylose to ethanol. Yeasts and most other fungi first reduce xylose to xylitol using xylose reductase, EC 1.1.1.21 (XR). This enzyme has strong preference for NADPH as a co-factor. In the following step, xylitol is oxidized to xylulose with a strictly NAD-dependent xylitol dehydrogenase, EC 1.1.1.9 (XDH) [2]. The difference in cofactor specificity in the first steps for xylose metabolism results in cofactor imbalance with reduced ethanol production and accumulation of xylitol [3-8]. Xylitol production can be reduced by metabolic engineering approaches directed to optimize the expression levels of XR and XDH [9-12], by changing the cofactor specificity of XR from NADPH to NADH [13,14], or by modifying the redox metabolism of the host cell [15-17]. Another approach used to bypass redox imbalance during xylose fermentation relies on the expression of fungal or bacterial xylose isomerase, EC 5.3.1.5 (XI) thereby directly converting xylose to xylulose with no requirement for NADPH or NADH as cofactors [18,19]. In the subsequent step, xylulose conversion can be further streamlined through the overexpression of xylulokinase, EC 2.7.1.17 (XK) (the third enzyme in the xylose metabolism that converts xylulose to xylulose-5-phosphate). The C5 sugar, xylulose-5-phosphate, is the entry point to the pentose phosphate pathway and into central metabolism. The overexpression of xylulokinase has been shown to enhance both aerobic and anaerobic xylose utilization in XR-XDH – as well as XI-carrying strains [5,20]. Overexpression of XK is necessary to overcome the naturally low expression level of this enzyme [3,5] and has been shown to result in more efficient conversion of xylose to ethanol [5,21].

The thermotolerant methylotrophic yeast *Hansenula polymorpha *is capable for alcoholic fermentation of xylose at elevated temperatures (45 – 48°C) [22-24]. Such property of the yeast defines it as a good candidate for use in efficient process of simultaneous saccharification and fermentation (SSF). This process combines enzymatic hydrolysis of lignocellulosic material with subsequent fermentation of released sugars in the same vessel. Among the major advantages for this yeast using are (i) well developed methods of molecular genetics and (ii) availability of genome sequence for the type strain CBS4732 [25,26].

In this article, we describe the construction of a recombinant *H. polymorpha *strain overexpressing the modified XR (K341R N343D) together with native XDH and XK on a *Δxyl1 *background. Xylose consumption, ethanol and xylitol production of this strain in comparison with those of strains overexpressing the native XR, XDH and XK are presented.

## Materials and methods

### Strains and media

Yeast strains *H. polymorpha *CBS4732 (*leu2-2*) [27], *Δxyl1 *[23] and transformants listed in Table 1 were grown on YPD (0.5% yeast extract, 1% peptone, 2% glucose) or minimal medium (0.67% YNB without amino acids, 4% xylose or 2% glucose) at 37°C. For the CBS4732s strain leucine (40 mg L^-1^) was supplemented into the medium. For selection of yeast transformants on YPD, 130 – 150 mg L^-1 ^of zeocin or 0.5 – 0.6 mg L^-1 ^of G418 were added.

**Table 1:** Strains and plasmids used in the present investigation.

Strains	Genotype	Reference
CBS4732	*leu 2-2*	[27]
*Δxyl1*	*Δxyl1::LEU2Sc*	[23]
XRn	*Δxyl1*, pX1N-Z *(GAPp-XYL1-AOXt)*	This study
XRm	*Δxyl1*, pX1M-Z *(GAPp-XYL1mod-AOXt)*	This study
XRn/XDH	*Δxyl1*, pX1N-Z-X2 *(GAPp-XYL1-AOXt, GAPp-XYL2-XYL2t)*	This study
XRm/XDH	*Δxyl1*, pX1M-Z-X2 *(GAPp-XYL1mod-AOXt, GAPp-XYL2-XYL2t)*	This study
XRn/XDH/XK	*Δxyl1*, pX1N-Z-X2 *(GAPp-XYL1-AOXt, GAPp-XYL2-XYL2t)*, pGLG61/HpXYL3 (*GAPp-XYL3-AOXt*)	This study
XRm/XDH/XK	*Δxyl1*, pX1M-Z-X2 *(GAPp-XYL1mod-AOXt, GAPp-XYL2-XYL2t)*, pGLG61/HpXYL3 (*GAPp-XYL3-AOXt*)	This study

The *E. coli *DH5α strain (Φ80d *lacZ*ΔM15, *recA*1, *endA*1, *gyrA*96, *thi*-1, *hsdR*17(r_K_^-^, m_K_^+^), *supE*44, *relA*1, *deoR*, Δ(*lacZYA-argF*)U169) was used as a host for propagation of plasmids. Strain DH5α was grown at 37°C in LB medium as described previously [28]. Transformed *E. coli *cells were maintained on a medium containing 100 mg L^-1 ^of ampicillin.

### Molecular-biology techniques

Standard cloning techniques were used as described [28]. Genomic DNA of *H. polymorpha *was isolated using the Wizard^® ^Genomic DNA Purification Kit (Promega, Madison, WI, USA). Restriction endonucleases and DNA ligase (Fermentas, Vilnius, Lithuania) were used according to the manufacturer specifications. Plasmid isolation from *E. coli *was performed with the Wizard^® ^*Plus *SV Minipreps DNA Purification System (Promega, Madison, WI, USA). PCR-amplification of the fragments of interest was done with Platinum^® ^*Taq *DNA Polymerase High Fidelity (Invitrogen, Carlsbad, CA, USA) according to the manufacturer specification. PCRs were performed in GeneAmp^® ^PCR System 9700 thermocycler (Applied Biosystems, Foster City, CA, USA). Transformation of the yeast *H. polymorpha *by electroporation was carried out as described previously [29].

### Plasmid construction

**Figure 1:**
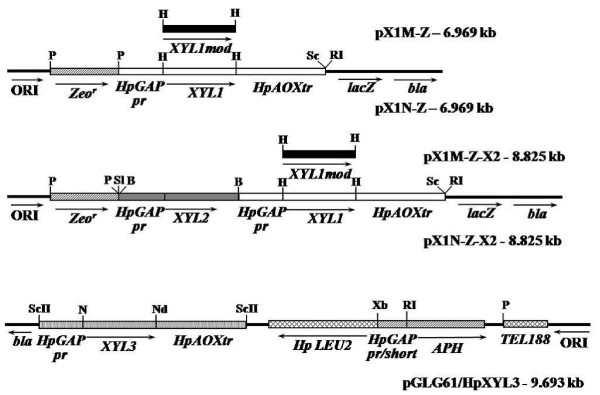
**Linear schemes of the plasmids used in this study: pX1M-Z, pX1N-Z, pX1M-Z-X2, pX1N-Z-X2 and pGLG61/HpXYL3.** Expression cassettes pr*GAP*-*XYL1*-tr*AOX*, pr*GAP*-*XYL2*-tr*XYL2 *and pr*GAP*-*XYL3*-tr*AOX *are shown as white, gray and doted boxes, respectively. The modified version of *XYL1 *ORF is shown as black box. The zeocin resistance gene (*Zeo*^r^) and geneticin resistance gene (*APH*), linked to an impaired constitutive gene promoter, encoding glyceraldehydephosphate dehydrogenase (*GAP*) are designated with the hatched lines. *H. polymorpha LEU2 *gene and the telomeric region (TEL188) [30] as an autonomously replicating sequence are shown as cross-hatched lines. Origin of replication ORI and ampicillin resistance gene (*bla*) – arrows. Restriction sites: P, *Pst*I; H, *Hind*III; S, *Sac*I; Sl, *Sal*I; B, *Bam*HI; ScII, *Sac*II; Xb, *Xba*I; RI, *Eco*RI; NdI, *Nde*I; N, *Not*I.

Recombinant plasmids pX1N-Z and pX1M-Z bearing native and modified version of XR, respectively, were constructed on the basis of the plasmid pUC57 (Fermentas, Vilnius, Lithuania). *Bam*HI/*Sac*I fragment with the *HpGAP *promoter and *HpAOX *terminator from the plasmid pKO8-GAPpr [23] was cloned into the *Bam*HI-*Sac*I digested plasmid pUC57 after elimination of restriction sites for *Nde*I and *Hind*III. The resulting plasmid restriction sites *Nde*I and *Not*I located between the *HpGAP *promoter and *HpAOX *terminator were removed, and a unique restriction site for *Hind*III produced. The ORF of *XYL1 *was PCR-amplified from genomic DNA of CBS4732 using pair of primers HpX1for (CCC AAG CTT ATG CAC ACG CAG ATT AGC AAA AAT CTT G) and HpX1rev (CGC AAG CTT TTA GAT AAA GGT TGG AAT TTC GTT CCA GGT CC) and cloned into the *Hind*III site to create expression cassette pr*GAP*-*XYL1*-tr*AOX *(restriction sites are underlined in all primers). Modification of XR gene was performed via overlap PCR. The pairs of primers HpX1Mfor (CAT CTT GGT CAT TCC AA**G **GTC C**G**A CCA AAA GGA GAG ACT G) and HpX1Mrev (CAG TCT CTC CTT TTG GT**C **GGA C**C**T TGG AAT GAC CAA GAT G) were used to produce K341 → R and N343 → D substitutions in resulting modified XR (mismatched bases for the mutation are shown in bold). Primers HpX1for and HpX1rev were used for cloning of modified version of XR gene as described above for the native gene. The yeast selective marker conferring resistance to zeocin was PCR-amplified from the plasmid pPICZB (Invitrogen) using a pair of primers: Ko58 (CGG GGT ACC TG CAG ATA ACT TCG TAT AGC ATA C) and Ko59 (CGG GGT ACC TG CAG TAA TTC GCT TCG GAT AAC) and cloned into the *Pst*I linearzed vectors to create pX1N-Z or pX1M-Z (Figure 1).

The *H. polymorpha XYL2 *gene with own terminator and the *HpGAP *promoter were amplified from the genomic DNA of CBS4732 using the corresponding pairs of primers Ko134 (GAT AAT ATA GAA ACA AAA TGA ACA ATC CTT CTG CTG)/Ko133 (ACA GGA TCC ATC CAT GAG AAA CG) and L1 (CTC GGA TCC CAA TTA TCA TTA ATA ATC)/Ko135 (CAG CAG AAG GAT TGT TCA TTT TGT TTC TAT ATT ATC). Primers L1 and Ko133 were used to obtain the fragment containing the *XYL2 *gene with own terminator driven with the *HpGAP *promoter by the overlap PCR. This fragment was cloned into the *Bam*HI linearized plasmids pX1N-Z i pX1M-Z, resulting in the recombinant constructs pX1N-Z-X2 and pX1M-Z-X2, respectively (Figure 1).

The expression cassette containing pr*GAP*-*XYL3*-tr*AOX *was obtained as *Sac*II restriction fragment from the plasmid pKO8/GAP/HpXYL3 [24] and cloned into the *Sac*II linearized plasmids pGLG61 [30]. The resulting plasmid was designated pGLG61/HpXYL3 (Figure 1). The accuracy of constructed plasmids was verified by sequencing. Constructed plasmids are presented in Table 1.

### Biochemical methods

The XR activity in cell extracts was determined spectrophotometrically at 37°C. The XR assay mixture contained: Tris-HCl buffer (pH 7.0) 100 mM, NADPH 0.15 mM and xylose 350 mM. The reaction was started with cell extract addition [3]. To evaluate K_M _towards NADPH or NADH, XR activities were measured with four different concentrations of cofactors 20, 50, 100 and 150 μM in triplicate.

The XDH activity in cell extracts was determined spectrophotometrically at 37°C. The XDH assay mixture contained: Tris-HCl buffer (pH 8.8) 100 mM, MgCl_2 _10 mM, NAD 3 mM and xylitol 300 mM. The reaction was started with cell extract addition [3].

The XK activity in cell extracts was determined spectrophotometrically at 37°C as described before [31], with some modifications. The XK assay mixture contained: Tris-HCl buffer (pH 7.8) 50 mM, MgCl_2 _5 mM, NADH 0.2 mM, phosphoenolpyruvate 1 mM, D-xylulose 8.5 mM, lactate dehydrogenase (EC 1.1.1.27) (Fluka, St. Louis, MO, USA) 10 U, pyruvate kinase (EC 2.7.1.40) (Fluka, St. Louis, MO, USA) 0.05 U, and ATP 2 mM. The reaction was started with addition of cell extract. For the XK assay, a blank without pyruvate kinase and lactate dehydrogenase was used to minimize interference of XDH activity in *H. polymorpha*.

All assay experiments were repeated at least twice.

### Analyses

Cells of transformants were grown in 100 ml of YPX medium (1% yeast extract, 2% peptone, 4% xylose) in Erlenmeyer flasks (bottle size – 300 ml) for 2 days and then inoculated into the 40 ml of YNB medium with 12% xylose in 100 ml Erlenmeyer flasks. Fermentation was carried out at the temperature of 48°C with limited aeration (140 revolutions × min^-1^). Concentrations of ethanol in medium were determined using alcohol oxidase/peroxidase-based enzymatic kit "Alcotest" [32]. Concentrations of xylitol in medium were determined enzymaticaly as described earlier (Enzymatic determination of D-sorbitol and xylitol, R-Biopharm GmbH, Darmstadt, Germany) with slight modifications. Nitrotetrazolium Blue (NTB) 12 mM and phenazine methosulfate 15 mM were used instead iodonitrotetrazolium chloride and diaphorase, respectively. The absorbance of the reduced NTB was measured at 570 nm. Concentrations of xylose from fermentation in mineral medium were analyzed by chemical method as described before [33]. The biomass was determined turbidimetrically in a FEK-56M photoelectric colorimeter (cuvette, 3 mm, light filter no. 6) with gravimetric calibration.

Experiments were performed at least twice.

## Results

### Engineering of XR

**Figure 2:**
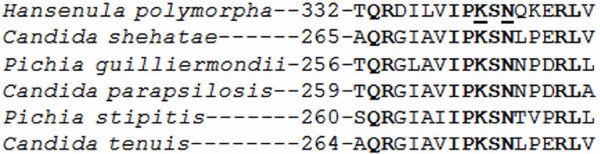
**Alignment of XRs cofactor binding site sequences from several xylose-utilizing yeasts against the *H. polymorpha XYL1 *sequence.** Conserved sequences are in bold. Underlined amino acids were changed (K → R and N → D).

To improve alcoholic fermentation of xylose and decrease xylitol formation, XR of *H. polymorpha *was subjected to site-specific mutagenesis to reduce its affinity for NADPH. The amino acid sequence of cofactor binding site of *H. polymorpha *XR shows strict homology to the corresponding site of other xylose-utilizing yeasts (Figure 2). Our aim was to substitute lysine and asparagine for arginine and aspartic acid at amino-acid positions 341 and 343, respectively. These substitutions resemble those developed for successful modification of XR cofactor specificity in *Candida tenuis *[34].

### Strain construction

To generate strains with overexpression of native or modified versions of XR or strains with simultaneous overexpression of native or modified XR together with XDH, the *H. polymorpha Δxyl1 *[23] strain was transformed with *Sac*I linearized plasmids pX1N-Z and pX1M-Z or pX1N-Z-X2 and pX1M-Z-X2, respectively. The transformants were grown on YPD medium supplemented with zeocin. The presence of expression cassettes in the transformants was examined by PCR using corresponding primers. To express the XK, the recombinant plasmid pGLG61/HpXYL3 was transformed into the recipient strain *H. polymorpha *overexpressing native or modified versions of XR and XDH. The transformants were grown on YPD medium in the presence of increasing concentrations of G418 addition. The highest concentration of G418, which allows the transformants to grow, was 0.4 mg × ml^-1^. Colonies able to grow on the selective medium appeared after 3 days of incubation with frequency of up to 20 transformants × mg^-1 ^DNA. The transformants were stabilized by cultivation in non-selective media for 10–12 generations with further shifting to the selective media with G418. The presence of recombinant *XYL3 *gene driven by the *HpGAP *promoter in genomic DNA of stable transformants was confirmed by PCR. As pGLG61-based plasmids promote multiple integration into the genome of recipient strains [30], constructed strains were examined by Southern hybridisation to select recombinant strains with equal amount of XK expression cassette. The strains bearing 3 copies of XK expression cassettes were selected (data not shown). Constructed yeast strains are represented in Table 1.

### Biochemical analysis of constructed strains

Biochemical properties of XR in one of constructed recombinant strains (designated XRm) were studied. Specific activities of XR (with both cofactors NADPH and NADH), XDH and XK as well as affinities of native XR (XRn) and engineered XRm were measured (Table 2). XRm was characterized by K_M _of 152 μM for NADPH using xylose as a substrate, which is 17 times higher than the K_M _for NADPH of the native XR (9 μM). K_M _of engineered XRm for NADH remained nearly unchanged (112 μM). Specific activity of XR with NADPH in the XRm strain decreased 9.1 times when compared with the strain overexpressing native XR. Specific activity of XR with NADH in both strains remained unchanged. Strains XRn/XDH and XRm/XDH with additional overexpressed XDH possessed two-fold increase in the specific activity of XDH as compared with the wild type strain. Overexpression of XK in strains XRn/XDH/XK and XRm/XDH/XK resulted in up to 2.4-fold increase in specific activity of XK as compared to CBS4732 (Table 2).

**Table 2:** XR, XDH, XK activities, ethanol and xylitol productivity of *H. polymorpha *transformants and control strain

Strain	Activity [U (mg protein^-1^)]				Productivity [mg (L h)^-1^]/[mg (L g h)^-1^]	
			
	XR		XDH	XK		
				
	NADPH/K_M _(μM)	NADH/K_M _(μM)			Ethanol	Xylitol
XRn	0.091 ± 0.005/9 ± 0.5	0.016 ± 0.001/100 ± 5.5	0.551 ± 0.035	-	6.3 ± 0.3/2.75 ± 0,11	3.6 ± 0.2/1.56 ± 0.1
XRm	0.01 ± 0.001/152 ± 8.4	0.014 ± 0.001/112 ± 6.5	0.504 ± 0.028	-	9.8 ± 0.4/4.45 ± 0.19	3.2 ± 0.2/1.45 ± 0.07
XRn/XDH	-	-	1.3 ± 0.07	-	12 ± 0.5/5.7 ± 0.3	3.1 ± 0.2/1.48 ± 0.07
XRm/XDH	-	-	1.485 ± 0.087	-	18.4 ± 0.9/8.76 ± 0.35	1.6 ± 0.1/0.76 ± 0.04
XRn/XDH/XK	-	-	-	0.375 ± 0.021	13.8 ± 0.6/6.4 ± 0.3	2.8 ± 0.1/1.3 ± 0.06
XRm/XDH/XK	-	-	-	0.366 ± 0.019	54.7 ± 2.7/21.9 ± 1.1	0.9 ± 0.04/0.36 ± 0.02
CBS4732	0.034 ± 0.025/7 ± 0.4	0.012 ± 0.001/85 ± 4.7	0.697 ± 0.049	0.156 ± 0.009	7.5 ± 0.4/3.3 ± 0.2	4.2 ± 0.2/1.8 ± 0.09

XRn – transformant bearing normal *XYL1 *gene; XRm – transformant bearing modified *XYL1 *gene (*XYL1m*).

- not determined

### Xylose fermentation

**Figure 3:**
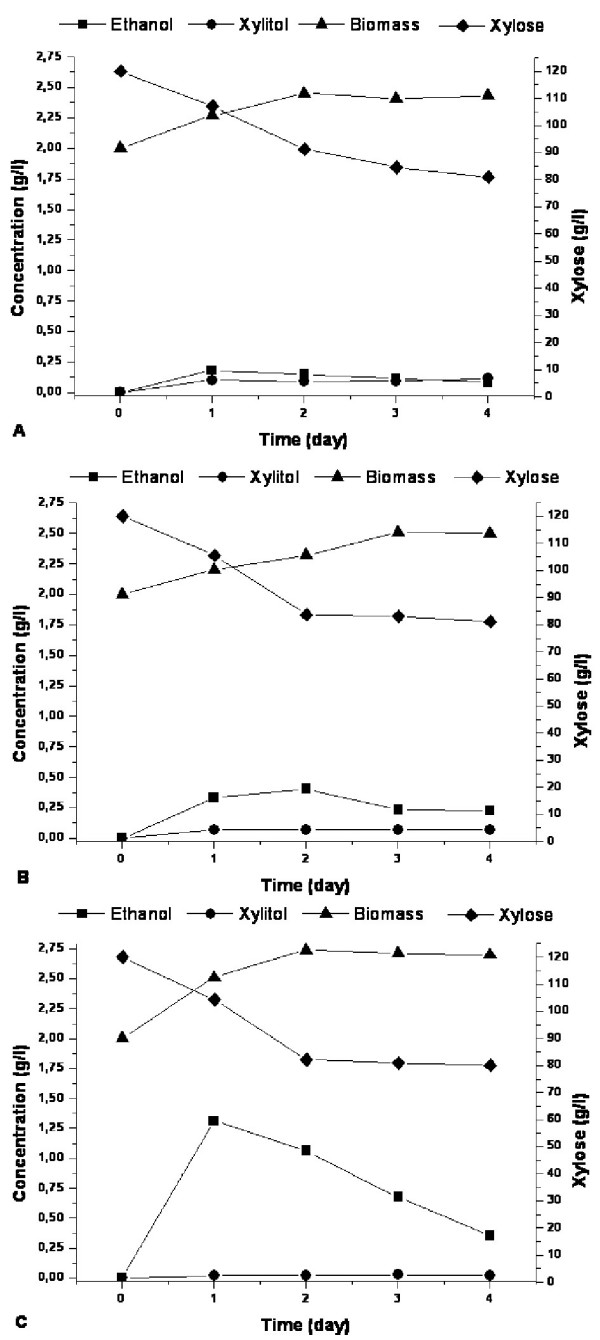
**Comparison of xylose fermentations at 48°C by CBS4732 (A), XRn/XDH/XK (B) and XRm/XDH/XK (C).** Symbols: ethanol (■), xylitol (●), biomass (▲) and xylose (◆).

Xylose fermentation by constructed strains was compared in batch cultures with limited aeration. A mineral medium containing xylose (12%) and an initial biomass concentration of 2 g (dry weight) × L^-1 ^were used. Results of ethanol and xylitol production by the constructed strains are shown in Table 2. Ethanol productivity of the XRm strain was 9.8 mg × (L × h)^-1^, which is 1.5- and 1.3-fold higher than the productivity of the XRn and the wild-type strain CBS4732, respectively. Xylitol production of these strains varied insignificantly. Ethanol productivity of the strain XRm/XDH (18.4 mg × (L × h)^-1^) was increased 1.5 and 2.4 times as compared to XRn/XDH and CBS4732, respectively. Strain XRm/XDH possessed 1.9- and 2.6-fold reduction in xylitol production compared with XRn/XDH and CBS4732 strains. Ethanol productivity of the strain XRm/XDH/XK (54.7 mg × (L × h)^-1^) was 4- and 7.3-fold higher compared to those of the strain XRn/XDH/XK (13.8 mg × (L × h)^-1^) and CBS4732 (7.5 mg × (L × h)^-1^). Xylitol production of the strain XRm/XDH/XK was significantly reduced to 0.9 mg × (L × h)^-1^, which is 3- and 4.7-fold lower than those of the XRn/XDH/XK and control strain, respectively. Representative fermentation profiles for the strains XRn/XDH/XK, XRm/XDH/XK and CBS4732 are shown in Figure 3. The specific ethanol and xylitol productivities per biomass at [mg (L g h)^-1^] of the recombinant and control strains were also calculated and represented at the Table 2. As biomass concentrations of the strains in all fermentation experiments were very similar, the values of comparison of specific ethanol and xylitol productivities per biomass between the strains are in the same level. The consumption of xylose by *H. polymorpha *strains during the fermentation is low. Ethanol produced in the initial stage of xylose fermentation is reutilized after 1–2 days of fermentation.

## Discussion

As described earlier [6], natural xylose-utilizing yeasts display alcoholic fermentation only when their native XR possessed NADH-linked activity. The XR of *H. polymorpha *belongs to enzymes with dual cofactor specificity, however NADPH is strongly preferred (>10-fold over NADH). In the present study, we focused our efforts on engineering an XR with increased K_M _for NADPH. Lysine and asparagine residues were substituted for arginine and aspartic acid, respectively, at the positions 341 and 343 in the cofactor binding site using the site-specific mutagenesis, based on sequence data of XR gene of *C. tenuis *[34]. The modified version of XR gene under control of the strong constitutive *HpGAP *promoter was overexpressed on the *Δxyl1 *background. This resulted in significant increase of K_M _for NADPH, while K_M _for NADH remained nearly unchanged. The results are in good agreement with reported features of modified XR from *C. tenuis *[34]. The constructed XRm strain showed a slight increase in ethanol productivity as compared to the wild type strain, while the overexpression of native XR had no positive effect. Xylitol production of these strains varied insignificantly. The cloned mutated XR gene results in significant lower specific activity with NADPH and in an increase in ethanol productivity of the host strain. To enable further improvement in ethanol production, XDH was expressed together with the modified XR. Overexpression of enzymes for initial two stages of xylose utilization pathway resulted in a 2.4-fold improvement of ethanol productivity accompanied by a 2.6-fold decrease in xylitol production.

In our previous work we developed *H*. *polymorpha *strains co-overexpressing *E. coli *XI and own XK. These strains were characterized by significant improvement of ethanol production during xylose fermentation [24]. In present study, the constructed strain XRm/XDH/XK overexpressing the modified XR together with XDH and XK is characterized with significant increase in ethanol productivity (up to 7.3 times) as compared to the wild type strain. It is worth mentioning that xylitol production by this strain is reduced considerably: 0.9 mg × (L × h)^-1 ^versus 4.2 mg × (L × h)^-1 ^by the wild type strain. Additional overexpression of XDH and XDH together with XK led to gradual increase in ethanol productivity and simultaneously decrease in xylitol production. It could be assumed that the initial stages of xylose utilization are limiting in alcoholic fermentation of xylose in *H. polymorpha*. The fermentation profiles of XRm/XDH/XK and XRn/XDH/XK are represented in Figure 3. The consumption of xylose by both constructed *H. polymorpha *strains and the wild type strain during the fermentation was rather low (Figure 3). This may suggest that xylose uptake in *H. polymorpha *is quite inefficient and corresponding genes coding putative xylose transporters should be cloned and overexpressed. In addition, bottlenecks downstream of XR cannot be excluded but this has to be determined. The fermentation profile revealed reutilization of synthesized ethanol. The reason of this phenomenon is not understood. We have isolated *H. polymorpha *2EtOH-mutant which is characterized by significant decrease in synthesized ethanol consumption (Ishchuk et al., unpublished). The molecular nature of the corresponding mutation, however, remains unknown.

Recombinant strains of *H. polymorpha *constructed in this study showed significant increase in ethanol productivity during high-temperature xylose fermentation. On the other hand, ethanol production from xylose is still very low as compared to the best current xylose fermenting strains [14,18,35]. Therefore, further efforts have to be applied to improve the xylose alcoholic fermentation in the thermotolerant yeast *H. polymorpha*.

## Conclusion

In the present work, co-overexpression of mutated form of XR (K341 → R N343 → D) together with native XDH and XK resulted in significant increase in ethanol productivity with simultaneous reduction of xylitol production during high-temperature xylose fermentation.

## Authors' contributions

OVD carried out the cloning, strains construction, evaluation of enzymes activity, fermentation experiments and co-drafted the manuscript. KVD participated in design of cloning and strain construction, performed enzyme assay and analyzed the date and wrote the manuscript. CAA participated in plasmids sequencing, commented and approved the manuscript. AYV participated in design and performance of cloning and commented the manuscript. AAS provided guidance and suggestions for experimental design and edited the manuscript. AYV and AAS conceived the presented study. All authors read and approved the final version of the manuscript.
